# Effect of the Two-Dimensional Magnetostrictive Fillers of CoFe_2_O_4_-Intercalated Graphene Oxide Sheets in 3-2 Type Poly(vinylidene fluoride)-Based Magnetoelectric Films

**DOI:** 10.3390/polym13111782

**Published:** 2021-05-28

**Authors:** Geunryeol Baek, Su-Chul Yang

**Affiliations:** Department of Chemical Engineering (BK21 FOUR Graduate Program), Dong-A University, Busan 49315, Korea; 1530278@donga.ac.kr

**Keywords:** magnetoelectric polymer composite films, poly(vinylidene fluoride), CoFe_2_O_4_-intercalated graphene oxides, two-dimensional magnetostrictive fillers

## Abstract

In the last decade, magnetoelectric (ME) polymer films have been developed by including zero-dimensional or one-dimensional magnetostrictive fillers in a piezoelectric polymer matrix. Existing reports on ME polymer films reveal that the shape of the magnetostrictive fillers is a critical determinant of the polymeric phase conformation, strain transfer between the piezoelectric and magnetostrictive phases, and dipole alignment in the films. In this study, to investigate the effect of two-dimensional (2D) magnetostrictive fillers on piezoelectric, magnetic, and magnetoelectric responses, 3-2 type ME films were prepared using CoFe_2_O_4_-intercalated graphene oxide (CFO-i-GO) fillers and poly(vinylidene fluoride) (PVDF) polymers. The 2D fillers of CFO-i-GO were hydrothermally synthesized by CFO intercalation into the interlayers of GO sheets with different lateral sizes, which were controlled by ultrasonication treatment. It was found that the large-lateral-size GO (LGO), medium-lateral-size GO (MGO), and small-lateral-size GO (SGO) fillers in the PVDF-based ME films exhibited a lateral size effect on CFO intercalation, polymeric phase conformation, dipole alignment, and magnetoelectric responses. A maximum ME coefficient (*α*_ME_) of 3.0 mV/cm∙Oe was achieved with a strong linearity (r^2^) of 0.9992 at an off-resonance frequency (*f*) of 1 kHz and applied direct current (dc) magnetic field (*H*_dc_) of ± 1000 Oe. The 3-2 type polymer-based ME films with reliable ME responses have potential for use in high-feasibility ME devices for biomedical sensing applications.

## 1. Introduction

Since 2000, magnetoelectric (ME) composites have gradually attracted significant interest because of their unique characteristics as well as piezoelectric and magnetostrictive properties, which can be used in various applications, such as high-sensitivity magnetic sensors, energy harvesters, memory devices, and even active cancer-targeting/drug-delivery systems [1,2,3,4,5,6]. The ME effect in multiphase composites was defined as an induced electric polarization in the piezoelectric phase owing to the mechanical stress caused by the shape change of the magnetostrictive phase under a magnetic bias field.

(1) $$ME\;effect=\frac{strain\;change}{magnetic\;field}\times \frac{electric\;polarization}{strain\;change}$$

Recently, polymer-based ME composites consisting of piezoelectric polymers and magnetostrictive materials have been developed using various structures, such as particulate composites with a 3-0 structure, laminate composites with a 2-2 structure, and electrospun nanofiber composites with porous structures. This is because piezoelectric polymers, such as poly(vinylidene fluoride) (PVDF), exhibit high flexibility, light weight, easy shaping, low-temperature processing, and biocompatibility, leading to their high feasibility for ME applications. Some studies on polymer-based ME composites have been reported over the past decade.

According to the literature reports on the 3-0 ME polymer composites, the ME coefficient (*α*_ME_) for poly(vinylidene fluoride-trifluoroethylene) [P(VDF-TrFE)]/Ni_0.5_Zn_0.5_Fe_2_O_4_ composites was 1.35 mV/cm·Oe at a resonance frequency (*f*_r_) of 40 kHz, and that for PVDF/CoFe_2_O_4_ ME composites was 11.2 mV/cm·Oe at a resonance frequency of 50 kHz; the slope was linear; therefore, the composites were suitable for highly sensitive magnetic sensors [7,8]. For P(VDF-TrFE)/Fe_3_O_4_ ME composites, the *α*_ME_ value was 0.8 mV/cm·Oe at 6 kHz. Spray-printed P(VDF-TrFE)/CoFe_2_O_4_ ME composites exhibited a maximum *α*_ME_ value of 21.2 mV/cm·Oe at 20 kHz. However, there is a limitation to the enhancement of the ME responses at the off-resonance frequency for the feasible applications of the 3-0 ME composites [9].

According to the reported literature on the 2-2 ME polymer laminates, poly(vinylidene fluoride-hexafluoropropylene) [P(VDF-HFP)]/Metglas ME composites exhibited *α*_ME_ of 12 V/cm·Oe under a low bias magnetic field (*H*_bias_) of 10 Oe [10]. By doping Al(NO_3_)_3_·9H_2_O in P(VDF-HFP)/Metglas ME laminates, ME responses were considerably improved via the induction of hydrogen bonds, with *α*_ME_ being 20 V/cm·Oe at *H*_bias_ of 4 Oe [11].

According to the reported literature on porous electrospun nanofiber ME composites, PVDF-HFP/Clay/NiFe_2_O_4_ ME composites exhibited an *α*_ME_ of 17 mV/cm·Oe with a filler content of 8 wt%. However, there is a limitation to applying a high electric field above 1 kV/cm during the electrical poling process, owing to the high leakage current of the magnetostrictive nanoparticles embedded in nanofibers [12].

In polymer ME films, PVDF has been commonly used as a piezoelectric phase with a strong dipole moment given by an electrically asymmetric *β*-phase with an all-trans (TTTT) configuration consisting of C–H and C–F bonds on opposite sides in the PVDF [13,14]. As a magnetostrictive phase in the polymer ME films, zero-dimensional (0D) or one-dimensional (1D) fillers have been used with spinel structure (AB_2_O_4_) compositions to enhance the *β*-phase in the piezoelectric phase [15,16,17]. The shape of the magnetostrictive fillers is a critical determinant of strain transfer to the piezoelectric phase, polymeric phase conformation, and leakage current, which directly affect the ME responses in polymer-based films [18,19].

**Figure 1:**
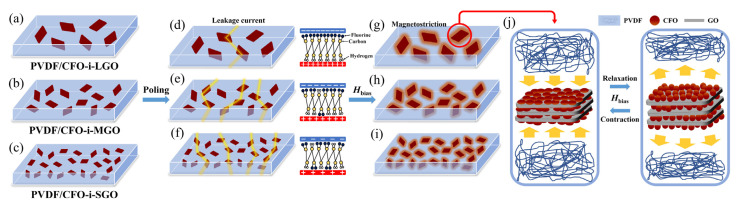
Schematic diagrams of the 3-2 type magnetoelectric (ME) films of (**a**) poly(vinylidene fluoride) (PVDF)/CFO-i-LGO (CoFe_2_O_4_-intercalated large-lateral-size graphene oxides), (**b**) PVDF/CFO-i-MGO(CoFe_2_O_4_-intercalated medium-lateral-size graphene oxides), and (**c**) PVDF/CFO-i-SGO(CoFe_2_O_4_-intercalated small-lateral-size graphene oxides); induced-leakage current in (**d**) PVDF/CFO-i-LGO, (**e**) PVDF/CFO-i-MGO, and (**f**) PVDF/CFO-i-SGO at a high electric field of 40 kV/mm; under an applied magnetic bias field (*H*_bias_) and active areas of magnetostriction in (**g**) PVDF/CFO-i-LGO, (**h**) PVDF/CFO-i-MGO, and (**i**) PVDF/CFO-i-SGO. (**j**) Conceptual illustration of *z*-axis magnetostriction in the 3-2 type ME films.

In this study, as a 3-2 type polymer-based ME film, PVDF/CoFe_2_O_4_-intercalated graphene oxide (CFO-i-GO) films were designed as shown in Figure 1. Two-dimensional (2D) magnetostrictive fillers of CFO-i-GO were synthesized by CFO intercalation into the interlayer of GO sheets; afterward, different lateral sizes of the fillers were controlled by ultrasonication time. To investigate the effect of lateral size on the magnetic, piezoelectric, and magnetoelectric properties, CFO intercalation, polymeric phase conformation, dipole alignment, and magnetoelectric responses were investigated using X-ray diffraction (XRD), energy-dispersive X-ray spectroscopy (EDX), vibrating sample magnetometer (VSM), Fourier-transform infrared spectroscopy (FT-IR), piezoelectric, and ME characterizations.

## 2. Experimental

### 2.1. Synthesis of Graphene Oxide Sheets

GO sheets were synthesized from graphite flakes using a modified Hummer’s method [20]. Graphite flakes (1.5 g, 99%, Asbury Carbons Inc., Asbury Park, NJ, USA) were added to a mixed solution consisting of 180 mL of H_2_SO_4_ (98%, Sigma-Aldrich, Saint Louis, MO, USA) and 20 mL of H_3_PO_4_ (85%, Sigma-Aldrich, Saint Louis, MO, USA) in 1L beaker for stable GO synthesis. Afterward, the mixed solution was stirred for 10 min. KMnO_4_ (9.0 g, 99%, Sigma-Aldrich, Saint Louis, MO, USA) was slowly added to the solution in an ice bath at 5 °C. Thereafter, the solution was vigorously stirred for 12 h on a hot plate at 45 °C until the solution color became dark brown. After the reaction, the solution was cooled to room temperature. Subsequently, the solution was poured into 200 mL of deionized (DI) water and stirred for 10 min. H_2_O_2_ (28.2 g, 30%, Daejung Chemicals and Metals Co., Siheung, Korea) was added to the solution, and the resulting mixed solution was stirred for 30 min. As a washing process, the mixture was centrifuged using 1 M HCl (30%, Daejung Chemicals and Metals Co., Siheung, Korea) aqueous solution. To obtain the GO sheets with different lateral sizes, the GO aqueous solutions were ultrasonicated 6 h (medium lateral size GO, MGO), and 24 h (small lateral size GO, SGO). The sheets obtained from solutions that were not ultrasonicated were considered large-lateral-size GO (LGO) sheets.

### 2.2. Synthesis of CoFe_2_O_4_-Intercalated Graphene Oxide

To prepare 2D-magnetostrictive fillers, 0.02 g of LGO, MGO, and SGO were dispersed in 10 mL of DI water. As a CFO precursor solution, 0.0743 g of Co(NO_3_)_2_∙6H_2_O (98%, Sigma-Aldrich, Saint Louis, MO, USA) and 0.2063 g of Fe(NO_3_)_3_∙9H_2_O (98%, Sigma-Aldrich, Saint Louis, MO, USA) were dispersed in 10 mL of DI water. Thereafter, the GO solutions were added to the CFO precursor solution, and the resulting solution was stirred for 30 min. An aqueous solution of 10 M NaOH (98%, Sigma-Aldrich, Saint Louis, MO, USA) was added to adjust the pH to 13, and the solution was stirred for 30 min. The mixed solutions were transferred into a 50 mL Teflon-lined stainless-steel autoclave, and a hydrothermal process was conducted at 180 °C for 20 h. After the hydrothermal reaction, three types of CFO-i-GO were obtained after washing with ethanol and DI water [21].

### 2.3. Fabrication of Magnetoelectric Films

Initially, to prepare 20 wt% PVDF/CFO-i-GO solution for fabrication of ME films, 2.587 g of PVDF powder (M_w_ ~534,000 g/mol, Sigma-Aldrich, Saint Louis, MO, USA) was dissolved in 10.667 g of N,N-dimethylformamide (DMF, anhydrous, 99.8%, Sigma-Aldrich, Saint Louis, MO, USA). Three types of CFO-i-GO fillers (0.08 g) were added to the PVDF solution while stirring it for 4 h. The PVDF/CFO-i-GO solutions were poured on a glass substrate and dried at room temperature for 12 h. After annealing the PVDF/CFO-i-GO at 200 °C for 10 min, hot pressing was conducted under 30 MPa at 150 °C for 1 h. The annealed PVDF/CFO-i-GO films exhibited an average thickness of 200 μm. Finally, electrical poling was conducted at 100 °C by applying a high electric field of 40 kV/mm for 3 h.

### 2.4. Characterization

The XRD patterns of graphite, LGO, MGO, SGO, CFO-i-GO, and PVDF/CFO-i-GO films were analyzed using an X-ray diffractometer (XRD, MiniFlex600, Rigaku, Tokyo, Japan) with CuKα (λ = 1.5406 Å) radiation. The morphologies of LGO, MGO, SGO, and CFO-i-GO were investigated by field emission scanning electron microscopy (FE-SEM, JSM-6700F, JEOL Ltd., Tokyo, Japan) with EDX (JSM-6700F, JEOL Ltd., Tokyo, Japan) at an accelerating voltage of 15 kV. The lateral sizes of the LGO, MGO, and SGO were measured by dynamic light scattering (DLS, Multiscope, K-One Nano Ltd., Seoul, Korea) and FE-SEM analysis. The magnetic hysteresis loops of the LGO, MGO, and SGO were obtained using a VSM (Model 7407-S, Lake Shore Cryotronics Inc., Westerville, OH, USA) at room temperature. The FT-IR spectra of the PVDF/CFO-i-GO films were obtained using an FT-IR spectrometer (Nicolet 380, Thermo Fisher Scientific Inc., Waltham, MA, USA) in attenuated total reflectance (ATR) mode in the range of 700–1500 cm^−1^. The ferroelectric hysteresis loops of the PVDF/CFO-i-GO films were measured at 10 Hz using a ferroelectric tester (Precision LC II, Radiant Technology Inc., Columbus, OH, USA). The piezoelectric charge constant (*d*_33_) of the PVDF/CFO-i-GO films was measured using a *d*_33_ meter (Model YE2730A, APC Inc., Mackeyville, PA, USA) after electrical poling. *α*_ME_ was investigated using a lock-in amplifier (SR860, Stanford Research Systems Inc., Sunnyvale, CA, USA) and Helmholtz coil.

## 3. Results and Discussion

**Figure 2:**
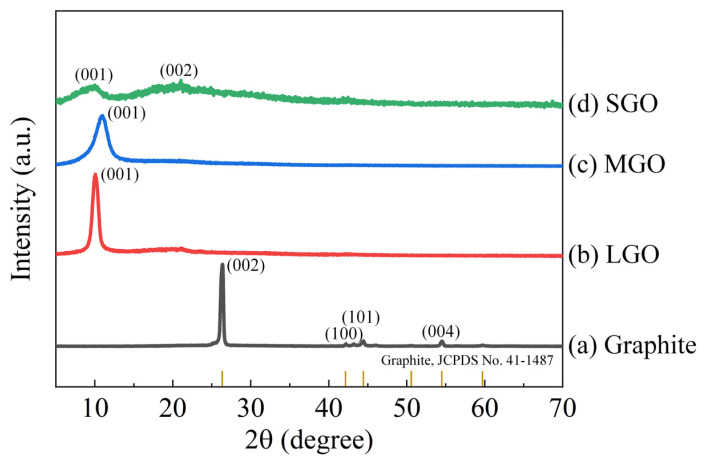
X-ray diffraction (XRD) patterns of (**a**) graphite, (**b**) LGO, (**c**) MGO, and (**d**) SGO.

The XRD patterns of graphite, LGO, MGO, and SGO were investigated, as shown in Figure 2. The XRD pattern exhibited clear peaks of (002), (101), (004), and (100), which corresponded to the graphite (JCPDS, No. 41-1487) [22]. After the conversion of the LGO sheets from graphite, a new XRD peak of (001) occurred at 2θ = 10.0°, corresponding to the interlayer distance (0.90 nm) of the LGO sheets. This demonstrates that oxygen-containing functional groups were introduced into the graphite [23]. The MGO sheets exhibited a broad XRD peak of (001), indicating the beginning of the collapse in the well-ordered interlayer of the LGO sheets during the ultrasonication for 6 h [24]. After excessive ultrasonication for 24 h, the broken interlayer of the SGO sheets was confirmed by the unclear XRD peaks of (001) and (002) [25,26].

**Figure 3:**
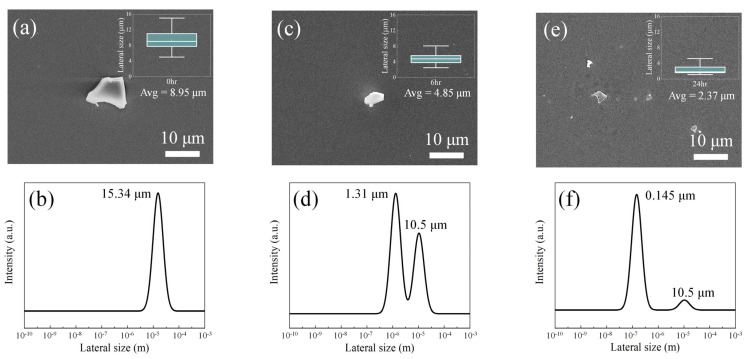
Representative scanning electron microscope (SEM) images of the (**a**) LGO, (**c**) MGO, and (**e**) SGO sheets. Inset data; box plots of the lateral size of the LGO, MGO, and SGO sheets. Dynamic light scattering (DLS) analysis of the lateral size distribution of the (**b**) LGO, (**d**) MGO, and (**f**) SGO sheets.

The morphology and lateral size of the GO sheets were investigated by FE-SEM and DLS analyses, as shown in Figure 3. The GO sheets exhibited a flake form with an average lateral size of 8.95 μm (LGO), 4.85 μm (MGO), and 2.37 μm (SGO), as observed from the inset box plots that were prepared by the size measurement of 20 specimens for each GO sheet. Notably, the average lateral size of the GO sheets and the size deviation decreased as the sonication time increased. The DLS analysis revealed that the sonicated GO sheets of MGO (6 h) and SGO (24 h) exhibited two peaks, illustrating sonication-dependent destruction, while the LGO sheets exhibited one peak without any destruction. As the sonication time increased, the peak split broadened, and the peak intensity at the small lateral sizes increased simultaneously. Furthermore, the LGO and SGO sheets respectively exhibited one lateral size of 15.34 μm and dominant lateral size of 0.145 μm; however, the MGO sheets were composed of two different lateral sizes: 1.31 and 10.5 μm.

**Figure 4:**
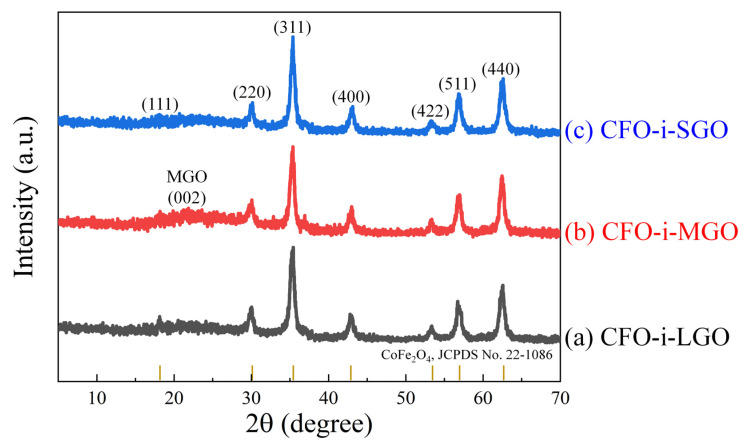
XRD patterns of the two-dimensional (2D) magnetostrictive fillers of (**a**) CFO-i-LGO, (**b**) CFO-i-MGO, and (**c**) CFO-i-SGO.

After magnetostrictive CFO intercalation into the interlayer of the GO sheets, XRD patterns were investigated to confirm the spinel crystal structure (AB_2_O_4_) and magnetostrictive material intercalation, as shown in Figure 4. The magnetostrictive fillers of CFO-i-LGO, CFO-i-MGO, and CFO-i-SGO exhibited clear peaks of (111), (220), (311), (440), (422), (511), and (440), which represent the cubic spinel structure of CoFe_2_O_4_ (JCPDS, No. 22-1086). The XRD patterns of the CFO-i-LGO and CFO-i-MGO fillers revealed that the main peak of (001) in the LGO and MGO sheets disappeared, which was due to the successful CFO intercalation into the interlayer of the LGO and MGO sheets [27]. This suggests that Co^2+^ and Fe^3+^ ions were intercalated into the interlayer of the GO sheets owing to electrostatic interaction between the negatively charged GO and positively charged metal ions, resulting in the crystal growth of CFO particles into the interlayer of the GO sheets [28,29]. However, in the CFO-i-SGO fillers, there might be few CFO intercalations into the interlayer of the SGO sheets, which was attributed to the destructive interlayers (Figure 2d) due to excessive sonication.

**Figure 5:**
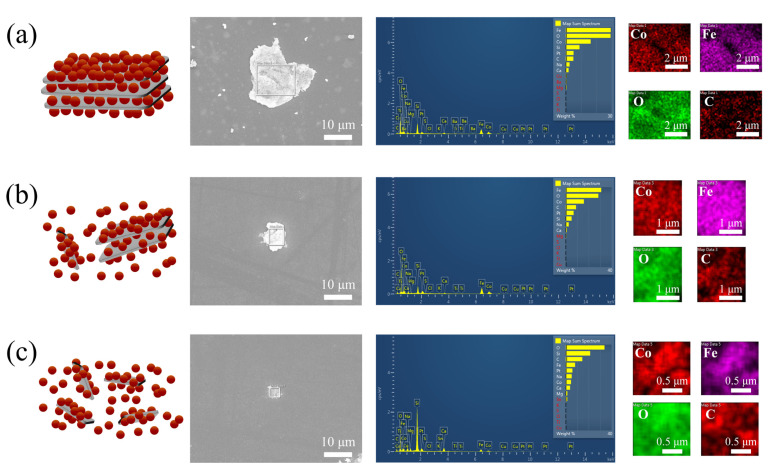
Schematic illustrations, representative SEM images, and EDX analysis of the 2D magnetostrictive fillers of (**a**) CFO-i-LGO, (**b**) CFO-i-MGO, and (**c**) CFO-i-SGO.

The EDX analysis of CFO-i-LGO, CFO-i-MGO, and CFO-i-SGO fillers was performed to determine the CFO amount in each GO sheet by calculating the Co/C weight ratio (Table 1). The magnetostrictive fillers exhibited Co/C weight ratios of 3.26 (CFO-i-LGO), 1.756 (CFO-i-MGO), and 0.319 (CFO-i-SGO). A high weight ratio of Co/C was achieved from CFO-i-LGO owing to sufficient CFO intercalation into the interlayer of the LGO sheets and CFO growth on the large sheet surface, as shown in the schematic illustration in Figure 5a. A medium weight ratio of Co/C was achieved from CFO-i-MGO owing to insufficient CFO intercalation into the small collapsed interlayer (Figure 2c) of the MGO sheets, as shown in the schematic illustration in Figure 5b. A low weight ratio of Co/C was achieved from CFO-i-SGO by a dominant CFO coating onto the SGO sheets with few CFO intercalations due to the almost destroyed interlayer (Figure 2d) of the SGO sheets, as shown in the schematic illustration of Figure 5c. The results implied that the CFO-intercalated GO fillers would provide effective magnetostriction along the *z*-axis direction in the ME films owing to the minimization of strain transfer loss between the magnetostrictive filler and piezoelectric polymer [30].

**Figure 6:**
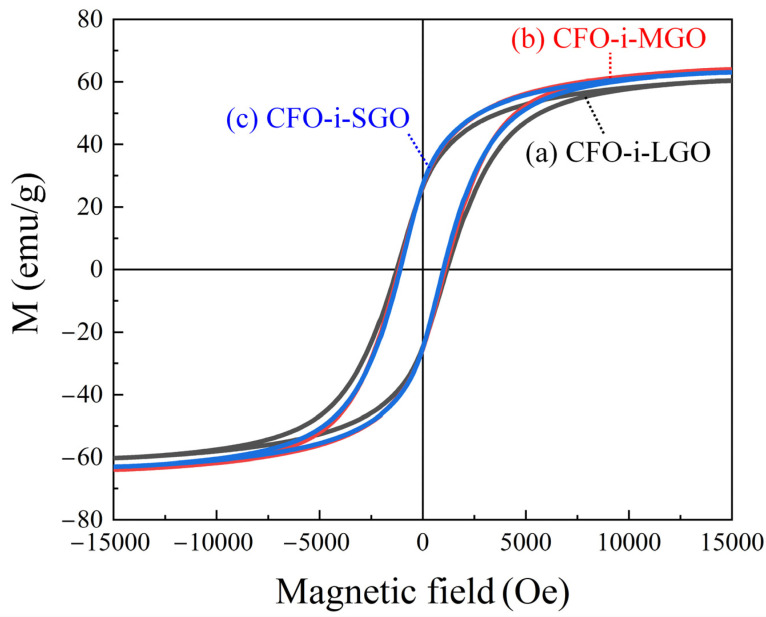
*M-H* hysteretic curves of the 2D magnetostrictive fillers of (**a**) CFO-i-LGO, (**b**) CFO-i-MGO, and (**c**) CFO-i-SGO.

The *M-H* loops of CFO-i-LGO, CFO-i-MGO, and CFO-i-SGO fillers were investigated with saturation magnetization (*M*_s_), remnant magnetization (*M*_r_), and coercive field (*H*_c_), as shown in Figure 6. Table 2 shows slight differences of ∆*M*_s_ = 5.7% and ∆*M*_r_ = 3.1% from the three types of fillers because there might be independent CFO particles, except for the CFO in the GO interlayer and the CFO on the GO sheet surface. Conversely, with a decrease in the lateral size of the fillers, *H*_c_ continuously decreased with ∆*H*_c_ = 12.85%, from 1256.81 Oe (CFO-i-LGO) to 1095.28 Oe (CFO-i-SGO), which might be caused by the free spin movement from the independent CFO particles compared to the intercalated CFO in the GO sheets [31].

**Figure 7:**
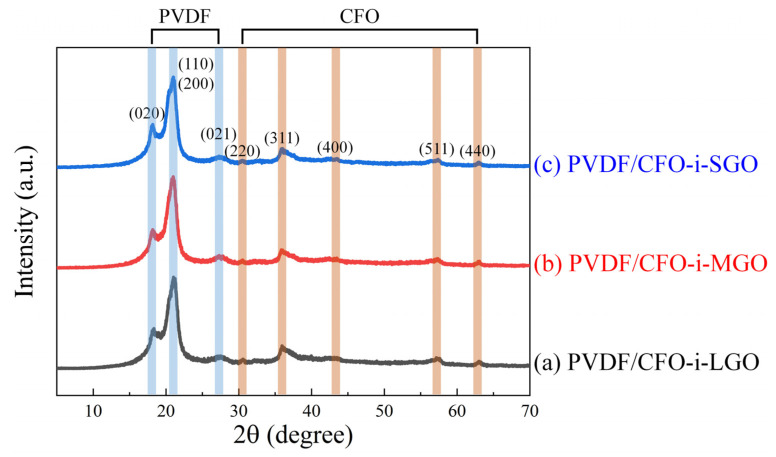
XRD patterns of the 3-2 type ME films of (**a**) PVDF/CFO-i-LGO, (**b**) PVDF/CFO-i-MGO, and (**c**) PVDF/CFO-i-SGO.

After preparing the ME films consisting of 3 wt% 2D-magnetostrictive fillers in a piezoelectric polymer matrix, the crystalline phases of PVDF and CFO-i-GO were investigated by XRD analysis, as shown in Figure 7. The ME films of PVDF/CFO-i-LGO, PVDF/CFO-i-MGO, and PVDF/CFO-i-SGO exhibited *α*-phase conformation from the (020), (110), and (021) peaks and *β*-phase conformation from the (110) and (200) peaks. [32,33,34] The XRD patterns showed weak peaks of (220), (311), (400), (511), and (440), representing a small amount of CFO in all the ME films.

**Figure 8:**
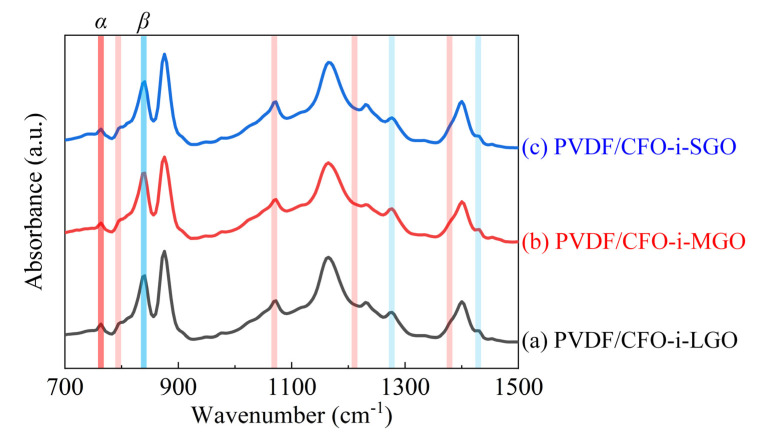
Fourier-transform infrared (FT-IR) spectra of the 3-2 type ME films of (**a**) PVDF/CFO-i-LGO, (**b**) PVDF/CFO-i-MGO, and (**c**) PVDF/CFO-i-SGO.

To calculate the *β*-phase content, the FT-IR spectra of the ME films were characterized in the range of 700–1500 cm^−1^, as shown in Figure 8. All the ME films exhibited prominent *β*-phase peaks at 840, 1275, and 1431 cm^−1^, while the α-phase peaks at 763, 795, 975, 1209, and 1383 cm^−1^ were not visible [35]. The following equation was introduced for the quantitative analysis of the *β*-phase content of the ME films [36]:

(2) $$F_{\beta }=\frac{A_{\beta }}{\left(\frac{K_{\beta }}{K_{\alpha }}\right)A_{\alpha }+A_{\beta }}\times 100\%$$

where *F_β_* is the *β*-phase content in the ME films; *K_β_* (7.7 × 10^−4^ cm^2^ × mol^−1^) and *K_α_* (6.1 × 10^−4^ cm^2^ × mol^−1^) are the absorption coefficients at wavenumbers of 840 and 763 cm^−1^, respectively. *A_β_* and *A_α_* are the absorbances at wavenumbers of 840 and 763 cm^−1^, respectively.

As shown in Table 3, all ME films exhibited high *β*-phase contents above 74.59%. This was due to a sufficient hot-press effect on the *β*-phase conformation by a distance extension between the F–C–H groups in the piezoelectric polymer chain [37]. Furthermore, a slight *β*-phase deviation of 2.94% was confirmed with a maximum *β*-phase of 77.53 in the PVDF/CFO-i-MGO films with the proper confinement effect to suppress the shrinkage of the polymer chain and for the good dispersion of the 2D-fillers in the PVDF matrix [18,38]. The PVDF/CFO-i-LGO films exhibited a *β*-phase content of 75.63% due to the locally dispersed 2D-fillers in the PVDF matrix, resulting in a low active area on the confinement effect, even though a strong confinement effect can be expected from the large lateral size of the CFO-i-LGO fillers [39,40]. The PVDF/CFO-i-SGO films exhibited a *β*-phase content of 74.59%, as a result of the weak confinement effect owing to the small lateral size of the CFO-i-SGO fillers, although a large active confinement area was formed owing to the considerable dispersion of the 2D-fillers in the PVDF matrix.

**Figure 9:**
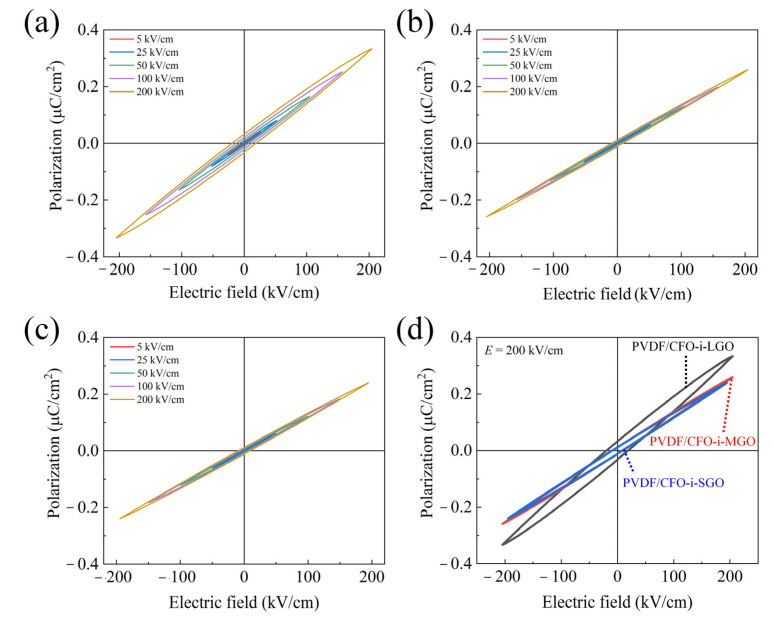
*P-E* hysteretic curves of the 3-2 type ME films of (**a**) PVDF/CFO-i-LGO, (**b**) PVDF/CFO-i-MGO, and (**c**) PVDF/CFO-i-SGO, and (**d**) *P-E* comparison under an applied electric field of 200 kV/cm.

The *P-E* loops of the ME films were investigated using saturation polarization (*P*_s_), remnant polarization (*P*_r_), and coercive field (*E*_c_) under an applied electric field of ± 200 kV/cm, as shown in Figure 9. Table 4 shows that the values of *P*_s_, *P*_r_, and *E*_c_ decreased as the lateral size of the magnetostrictive fillers decreased. A high *P*_s_ of 0.3333 μC/cm^2^ was achieved for the PVDF/CFO-i-LGO films, even though the PVDF/CFO-i-MGO films exhibited the maximum *β*-phase content, representing the active piezoelectric phase. This demonstrates that leakage current should be considered for high piezoelectric properties in the ME films because strong dipole alignment can be achieved under an intact electric field with a low leakage current [41,42]. Therefore, the PVDF/CFO-i-LGO films exhibited a small leakage current, which might induce high dipole alignment in the PVDF matrix. Furthermore, a low *P*_s_ of 0.2394 μC/cm^2^ was achieved from the PVDF/CFO-i-SGO films with a high leakage current and minimum *β*-phase content.

The effect of leakage current was critical during the direct poling at 100 °C under applied high direct current (dc) electric fields for 3 h. The electric strength and *d*_33_ of the ME films are listed in Table 5. With a decrease in the lateral size of the 2D-fillers, the allowable applied dc voltage decreased from 40 kV/mm (PVDF/CFO-i-LGO) to 25 kV/mm (PVDF/CFO-i-SGO); thus, the electric strength decreased, determining the piezoelectric properties of *d*_33_. The low electric strength of the PVDF/CFO-i-MGO and PVDF/CFO-i-SGO films might be limited by the independently existent small CFO particles (Figure 5a,b) in the films. Therefore, a maximum *d*_33_ of 14 pC/N was achieved from the PVDF/CFO-i-LGO film, illustrating a high electric strength of 40 kV/mm, high *P*_s_ of 0.3333 μC/cm^2^, and medium *β*-phase content of 75.63%.

**Figure 10:**
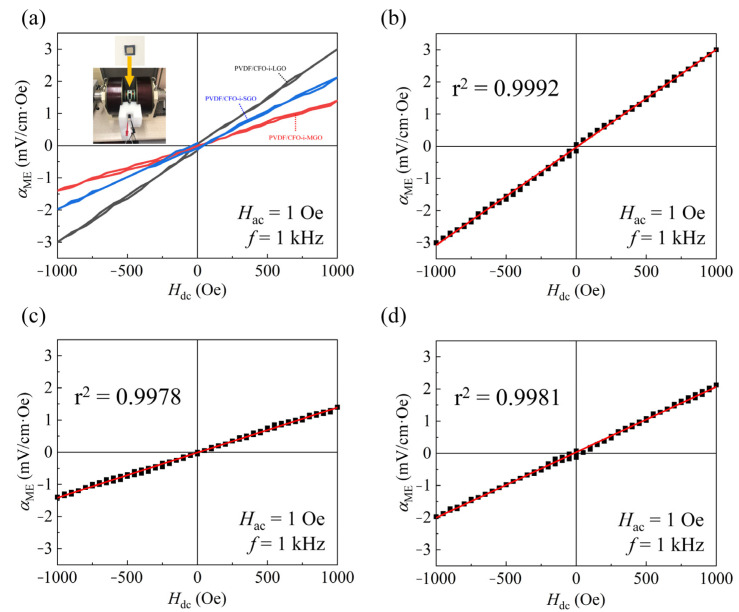
(**a**) Comparison of the ME responses in the 3-2 type ME films of PVDF/CFO-i-LGO, PVDF/CFO-i-MGO, and PVDF/CFO-i-SGO as a function of the dc magnetic field (*H*_dc_) under an applied ac magnetic field (*H*_ac_) of 1 Oe at an off-resonance frequency (*f*) of 1 kHz. Calculated ME linearity (r^2^) of (**b**) PVDF/CFO-i-LGO, (**c**) PVDF/CFO-i-MGO, and (**d**) PVDF/CFO-i-SGO.

To investigate the ME responses of the films, the *α*_ME_ was measured by applying an alternating current (ac) magnetic field (*H*_ac_) of 1 Oe at an off-resonance frequency (*f*) of 1 kHz under a dc magnetic field (*H*_dc_) of ± 1000 Oe, as shown in the inset of Figure 10a. As shown in Table 5, the ME values of *α*_ME_ are 3.0 mV/cm∙Oe (PVDF/CFO-i-LGO), 1.4 mV/cm∙Oe (PVDF/CFO-i-MGO), and 2.13 mV/cm∙Oe (PVDF/CFO-i-SGO). The results illustrate that a maximum *α*_ME_ of 3.0 mV/cm∙Oe was achieved from the PVDF/CFO-i-LGO films with effective strain transfer and high piezoelectric properties. The *α*_ME_ value of 2.14 mV/cm∙Oe for the PVDF/CFO-i-SGO films shows that the considerable dispersion of the magnetostrictive fillers in the piezoelectric matrix is important for high ME responses in the particulate ME films. Even though the PVDF/CFO-i-SGO films were found to exhibit relatively low values of *β*-phase, *P*_s_, electric strength, and *d*_33_ compared to those in the PVDF/CFO-i-MGO films, relatively high *α*_ME_ values were obtained owing to the enlarged active area (Figure 1i) in the PVDF/CFO-i-SGO films. In particular, the 3-2 type polymer-based ME films exhibited proportional ME responses with a linear behavior under an applied *H*_dc_ of ± 1000 Oe. To determine the sensing capability, the linearity of the ME films was calculated, and the r^2^ values were 0.9992 (PVDF/CFO-i-LGO), 0.9978 (PVDF/CFO-i-MGO), and 0.9981 (PVDF/CFO-i-SGO), greater than those reported in the literature [43]. Therefore, the 3-2 type polymer-based ME films with reliable ME responses have a high feasibility and can be used in ME devices for biomedical-sensing applications.

## 4. Conclusions

In this study, polymer-based ME PVDF/CFO-i-GO films with high feasibility were prepared as 3-2 type particulate ME films. In particular, different lateral size fillers of LGO, MGO, and SGO were prepared to investigate the effect of lateral size on CFO intercalation, polymeric phase conformation, dipole alignment, and magnetoelectric responses. The films exhibited maximum ME *α*_ME_ values of 3.0 mV/cm∙Oe (PVDF/CFO-i-LGO), 1.4 mV/cm∙Oe (PVDF/CFO-i-MGO), and 2.13 mV/cm∙Oe (PVDF/CFO-i-SGO) at 1 kHz. The maximum *α*_ME_ was achieved from the PVDF/CFO-i-LGO by optimizing the lateral size effect, resulting in effective strain transfer and high piezoelectric properties. Furthermore, the ME films exhibited linear ME behavior with strong r^2^ values of 0.9992 (PVDF/CFO-i-LGO), 0.9978 (PVDF/CFO-i-MGO), and 0.9981 (PVDF/CFO-i-SGO). The results suggest that the 3-2 type polymer-based ME films can be used in potential applications, such as non-contact biomedical sensors, for real-time health monitoring.

## Figures and Tables

**Table 1:** Amount of chemical composition in the 2D magnetostrictive fillers of CFO-i-LGO, CFO-i-MGO, and CFO-i-SGO confirmed by energy-dispersive X-ray spectroscopy (EDX) analysis.

2D-Fillers	Chemical Composition (wt%)				
	Co	Fe	O	C	Others
CFO-i-LGO	16.3	29.4	29.3	5.0	20.0
CFO-i-MGO	15.8	31.0	28.4	9.0	15.8
CFO-i-SGO	4.6	8.1	33.8	14.4	39.1

**Table 2:** Magnetic properties in the 2D magnetostrictive fillers of CFO-i-LGO, CFO-i-MGO, and CFO-i-SGO confirmed by vibrating sample magnetometer (VSM) measurement.

2D-Fillers	*M*_s_ (emu/g)	*M*_r_ (emu/g)	*H*_c_ (Oe)
CFO-i-LGO	60.33	26.23	1256.81
CFO-i-MGO	63.98	26.73	1148.48
CFO-i-SGO	63.03	27.08	1095.28

**Table 3:** Calculated *β*-phase content in the 3-2 type ME films of PVDF/CFO-i-LGO, PVDF/CFO-i-MGO, and PVDF/CFO-i-SGO.

ME Films	*β*-Phase Content (%)
PVDF/CFO-i-LGO	75.63
PVDF/CFO-i-MGO	77.53
PVDF/CFO-i-SGO	74.59

**Table 4:** Electrical properties in the 3-2 type ME films of PVDF/CFO-i-LGO, PVDF/CFO-i-MGO, and PVDF/CFO-i-SGO.

ME Films	*P*_s_ (μC/cm^2^)	*P*_r_ (μC/cm^2^)	*E*_c_ (kV/cm)
PVDF/CFO-i-LGO	0.3333	0.032	19.50
PVDF/CFO-i-MGO	0.2591	0.012	10.73
PVDF/CFO-i-SGO	0.2394	0.012	9.94

**Table 5:** Electric strength, piezoelectric, and ME properties in the 3-2 type ME films of PVDF/CFO-i-LGO, PVDF/CFO-i-MGO, and PVDF/CFO-i-SGO.

ME Films	Electric Strength (kV/mm)	∆*d*_33_ (pC/N)	*α*_ME_ (mV/cm·Oe)
PVDF/CFO-i-LGO	40	14	3.0
PVDF/CFO-i-MGO	28	7.5	1.4
PVDF/CFO-i-SGO	25	6	2.13

## Data Availability

Data are contained within this article.
